# Sensitivity of the Autonomic Nervous System to Visual and Auditory Affect Across Social and Non-Social Domains in Williams Syndrome

**DOI:** 10.3389/fpsyg.2012.00343

**Published:** 2012-09-14

**Authors:** Anna Järvinen, Benjamin Dering, Dirk Neumann, Rowena Ng, Davide Crivelli, Mark Grichanik, Julie R. Korenberg, Ursula Bellugi

**Affiliations:** ^1^Laboratory for Cognitive Neuroscience, The Salk Institute for Biological StudiesLa Jolla, CA, USA; ^2^Brain and Mind Laboratory, Department of Biomedical Engineering and Computational Science, Aalto University School of ScienceEspoo, Finland; ^3^Emotion and Social Cognition Laboratory, California Institute of TechnologyPasadena, CA, USA; ^4^Department of Psychology, Catholic University of the Sacred HeartMilan, Italy; ^5^The Brain Institute, The University of UtahSalt Lake City, UT, USA

**Keywords:** williams syndrome, affect, electrodermal activity, heart rate, facial expression, autonomic nervous system, psychophysiology

## Abstract

Although individuals with Williams syndrome (WS) typically demonstrate an increased appetitive social drive, their social profile is characterized by dissociations, including socially fearless behavior coupled with anxiousness, and distinct patterns of “peaks and valleys” of ability. The aim of this study was to compare the processing of social and non-social visually and aurally presented affective stimuli, at the levels of behavior and autonomic nervous system (ANS) responsivity, in individuals with WS contrasted with a typically developing (TD) group, with the view of elucidating the highly sociable and emotionally sensitive predisposition noted in WS. Behavioral findings supported previous studies of enhanced competence in processing social over non-social stimuli by individuals with WS; however, the patterns of ANS functioning underlying the behavioral performance revealed a surprising profile previously undocumented in WS. Specifically, increased heart rate (HR) reactivity, and a failure for electrodermal activity to habituate were found in individuals with WS contrasted with the TD group, predominantly in response to visual social affective stimuli. Within the auditory domain, greater arousal linked to variation in heart beat period was observed in relation to music stimuli in individuals with WS. Taken together, the findings suggest that the pattern of ANS response in WS is more complex than previously noted, with increased arousal to face and music stimuli potentially underpinning the heightened behavioral emotionality to such stimuli. The lack of habituation may underlie the increased affiliation and attraction to faces characterizing individuals with WS. Future research directions are suggested.

## Introduction

Understanding the underpinnings of human social behavior is of relevance to both typical development as well as neurodevelopmental disorders. Within this realm, the unusual social phenotype associated with Williams syndrome (WS) has brought this neurogenetic condition to the forefront of interest within the neuroscience community. Specifically, WS, caused by a contiguous deletion of 25–30 genes on chromosome 7q11.23 (Ewart et al., 1993; Korenberg et al., 2000), combined with a distinctive social profile, holds promise for discovering linkages between neurobiological, physiological, behavioral, and genetic systems that provide meaning to human social interaction. Individuals with WS demonstrate an unusually positive expression of affect, abnormally expressive language in narratives, increased attraction to and engagement with strangers, a propensity to direct eye contact, a relative strength in identifying and remembering faces, increased empathy, and an increased emotional reactivity to music combined with intriguing dissociations, which include overly friendly behavior with a difficulty in making friends, social fearlessness coupled with anxiety, and abundant positive affect with maladaptive behaviors (see Dykens, 2003; Levitin et al., 2004; Meyer-Lindenberg et al., 2006; Järvinen-Pasley et al., 2008; Martens et al., 2008; Riby and Porter, 2010; for reviews). Perhaps not surprisingly, in general terms, WS is associated with more competent processing of social as compared to non-social information (e.g., Martens et al., 2008), a remark that we recently extended into the realm of emotion processing of faces vs. non-social images (Järvinen-Pasley et al., 2010a).

Although the social affective characteristics have variably been described in WS for a decade, specifically the role of the underlying autonomic nervous system (ANS) function in the fascinating social phenotype remains largely unknown. This question is of significance for several reasons. First, individuals with WS have been described as demonstrating heightened emotional reactivity at the behavioral level (e.g., Tager-Flusberg and Sullivan, 2000; Levitin et al., 2004), and it is currently not understood whether this unusual sensitivity may play a role in regulating the reward value of social encounters for individuals with WS. Second, the examination of patterns of ANS sensitivity of individuals with WS to affective social and non-social stimuli within both visual and auditory in modalities may provide some clues regarding the nature of the diagnostically significant anxiety associated with the syndrome. Thus, the social behavior associated with WS comprises a panoply of distinct socially positive and maladaptive behaviors implicating the dysfunction of multiple neural circuits. As both electrodermal activity (EDA) and heart rate (HR) are amygdala-associated non-invasive and robust measures of autonomic function indexing sensitivity to social affective information at physiological levels (LeDoux, 2000; Adolphs, 2001; Laine et al., 2009), they provide useful tools for elucidating the underpinnings of the social-emotional phenotype associated with WS. Moreover, the physiological processes indexed by electrodermal responses and HR are regulated by the hypothalamic-pituitary axis (HPA) implicated in a variety of social behaviors across species (Pfaff, 1999; Goodson, 2005). More broadly, understanding the role of autonomic function to social tendencies is also of interest from standpoint of individual differences research, as the extraverted and introverted personality profiles have also been linked to individual differences in ANS functioning. Specifically, differences in arousal between extraverts and introverts have been linked to emotional experience and social behavior (Eysenck, 1967, 1994, 1997). Whereas extraverts seek more frequent and intense stimulation (e.g., social interaction) to raise their inherently low level of arousal to an optimal level, introverts tend to exhibit the opposite pattern. Thus, compared to extraverts, introverts are thought to be inherently more aroused and arousable (Stelmack, 1990; Smith, 1994); exhibit higher HR reactivity (Smith et al., 1995); higher skin conductance levels (SCL; Smith et al., 1986); greater phasic skin conductance response (SCR; Smith et al., 1990); and slower electrodermal habituation (Smith et al., 1995). Although some aspects of this theory have been questioned (see e.g., Beauducel et al., 2006, for a discussion), it is nevertheless of interest to explore how the WS social profile of anxiousness coupled with hypersociability and its ANS correlates fit in with this line of research, as some behavioral features resemble typical extraverted behavior. WS provides a special window into the underpinnings of social information processing due to it representing atypical genetic expression, linked to increased social and emotional behavior.

A handful of previous studies have explored electrodermal and/or HR activity in WS within the visual domain. In one study, Plesa Skwerer et al. (2009) presented individuals with WS, chronological age (CA)-matched typically developing (TD) individuals, and those with mental disabilities matched for language abilities, with dynamic facial expressions anger, disgust, fear, happiness, sadness, surprise, and neutral emotion, while SCR and HR were recorded. Emotionally neutral nature videos were also presented. The results indicated that relative to both control groups, WS was associated with hypoarousal in response to face stimuli, and these participants also showed more pronounced HR deceleration, interpreted as indexing heightened interest in such stimuli. In another study, Doherty-Sneddon et al. (2009) assessed changes in SCR while individuals with WS and CA-matched TD controls completed arithmetic tasks varying in both difficulty and the extent of eye contact with the experimenter. In another task, the extent of gaze aversion in relation to cognitive load was assessed. The results showed that while WS was associated with general hypoarousal and reduced gaze aversion, similar to the TD controls, their arousal level increased in response to face stimuli. The authors suggested that lower than typical arousal level may enable individuals with WS to hold face and eye gaze for prolonged periods of time, which may underlie their aberrant face and eye gaze processing strategies, and ultimately, some social features (see, e.g., Riby and Hancock, 2008, 2009). Plesa Skwerer et al. (2011) recently tested ANS responsivity as reflected in pupil dilation in individuals with WS contrasted with CA- and MA-matched control groups in response to viewing images with social vs. non-social content. The results showed that although all groups showed greater arousal to the social as compared to the non-social images, the WS group displayed decreased pupil dilation to faces expressing negative affect relative to the controls. It is also noteworthy that overall, WS was associated with decreased pupil dilation to the social stimuli, suggesting reduced arousal to social stimuli relative to the control groups. Finally, Riby et al. (2012) examined SCL reactivity in response to live and video-mediated displays of happy, sad, and neutral affect in individuals with WS, TD, and autism. The results showed that only live faces increased the level of arousal for those with WS and TD. Participants with WS displayed lower SCL as compared to the TD group, which the authors interpreted as suggesting hypoarousal in this group.

Taken together, the findings described above raise several questions about the role of ANS functioning within the WS social phenotype. The studies of Plesa Skwerer et al. (2009), Doherty-Sneddon et al. (2009), and Riby et al. (2012) will be considered here as they are of specific relevance to the current study due to them involving EDA and/or HR measures. In particular, the discrepant finding of hyporesponsivity to emotional face stimuli in Plesa Skwerer et al.’s (2009) study and the typical increase in arousal in response to faces in Doherty-Sneddon et al.’s (2009) and Riby et al.’s (2012) studies warrants further research. It is possible that the different nature of the paradigms used contributes to this seeming contradiction: namely, while the studies of Doherty-Sneddon et al. and Riby et al. involved a naturalistic social context with live face stimuli, Plesa Skwerer et al. (2009) used computer-delivered facial displays of affect. Differences in methodologies employed between the two studies may further account for some of the inconsistencies. To address these gaps, the current study will involve two analogous paradigms across the visual and auditory modalities, contrasting the processing of social (face/voice) with similar non-social (image of an object or scene/music) stimuli matched for emotion. The non-social contrast condition will allow us to determine the extent to which social stimuli are special for individuals with WS. Further, extending this line of research into the domain of auditory processing is important, as affective expressions typically are multimodal, and critical social information is provided both by a face and a voice. Albeit scarce, there is evidence of aberrant behavioral and neurobiological organization of socially relevant auditory processing in WS (see, e.g., Järvinen-Pasley et al., 2010b). It is noteworthy that in general, significantly less is known about the neural processing of aurally presented, as compared with visually presented affect (Adolphs, 2002), although both facial expressions and affective vocalizations comprise the two most frequently employed channels of communicating emotion in social interactions. Thus, a systematic study in which the same participants complete analogous visual and auditory tasks as is proposed here will elucidate such processes.

Given that the current paradigms were more similar in nature (i.e., not representing naturalistic social situations) to that of Plesa Skwerer et al. (2009) as compared to those of Doherty-Sneddon et al. (2009) and Riby et al. (2012), we hypothesized that relative to TD participants, individuals with WS would exhibit attenuated EDA and greater HR deceleration in response to affective social stimuli, while the opposite pattern would be predicted for the non-social stimuli (e.g., face vs. non-social images; human vocalizations vs. music). The opposite profiles across the social and non-social domains were predicted for the TD group.

## Experiment 1: Faces vs. Scenes: ANS Sensitivity to Visual Social and Non-Social Affective Stimuli

### Material and methods

#### Participants

Twenty-two individuals with WS (eight males) were recruited through a multicenter program based at the Salk Institute. For all participants, genetic diagnosis of WS was established using fluorescence *in situ* hybridization (FISH) probes for elastin (ELN), a gene invariably associated with the WS microdeletion (Ewart et al., 1993; Korenberg et al., 2000). In addition, all participants exhibited the medical and clinical features of the WS phenotype, including cognitive, behavioral, and physical features (Bellugi et al., 2000). Twenty-seven TD individuals (11 males) were recruited as controls; however, the data of four participants were excluded from the analyses due to perseveration, specifically, they responded to all non-social stimuli as “neutral.” This resulted in a final sample of 23 TD individuals (nine males) in the behavioral analysis. In subsequent psychophysiology analyses (different recordings than the behavioral data), identification accuracy was a factor controlled in our modeling approach, hence, this sample contained 20 individuals with WS (eight males; mean age = 27.57, SD = 7.70; the data from two participants with WS were excluded due to excessive recording artifact) and 27 TD participants (11 males; mean age = 21.40, SD = 4.32). The participants were screened for the level of education, and those with more than 2 years of college-level education were excluded from this study. Each participant was screened for current and past psychiatric and/or neurological problems, and only those deemed clinically asymptomatic were included in the study. All participants were of the same cultural background, i.e., American.

The participants’ cognitive functioning was assessed using the Wechsler Intelligence Scale. Participants under 16 years of age were administered the Wechsler Intelligence Scale for Children Third Edition (WISC-III; Wechsler, 1991), and those above 16 years of age were administered either the Wechsler Adult Intelligence Scale Third Edition (WAIS-III; Wechsler, 1997) or the Wechsler Abbreviated Scale of Intelligence (WASI; Wechsler, 1999). Participants were also administered the Benton Test of Facial Recognition (Benton et al., 1983), a perceptual face discrimination task. In addition, all participants were native English speakers, and gave written informed consent before participation. Written informed assent was also obtained from participants’ parents, guardians, or conservators. All experimental procedures complied with the standards of the Institutional Review Board at the Salk Institute for Biological Studies.

Table 1 shows the demographic characteristics of the final sample of participants with WS and TD. The participants differed in terms of CA [*t*(43) = 2.93, *p* = 0.005] with the WS group being older than the TD group. While the WS and TD groups did not differ significantly on the basis of the Benton Test standardized scores [*t*(43) = −1.48, *p* = 0.15], expectedly, the TD group scored significantly higher on VIQ, PIQ, and FSIQ (all *p* < 0.001). Pearson correlations exploring the potential contributions of CA, Benton Test standardized scores, VIQ, PIQ, and FSIQ to task performance showed that for those with WS, VIQ correlated positively with the identification of facial emotion [*r* (18) = 0.59, *p* = 0.009], while all other correlations failed to reach significance (all *p* > 0.11). No significant associations emerged for the TD group (all *p* > 0.12).

**Table 1:** **Mean characteristics of the participant groups (SD; range in parentheses) in Experiment 1**.

	CA (SD; range)	VIQ (SD; range)	PIQ (SD; range)	FSIQ (SD; range)	Benton SS (SD; range)
WS (*n* = 22)	27.3 (8.2; 13–46)	71 (8.3; 55–86)	63 (9.3; 53–72)	66 (7.5; 51–84)	93 (19.1; 55–129)
TD (*n* = 23)	21.5 (4.6; 18–41)	112 (9.76; 89–129)	113 (9.2; 89–127)	114 (7.91; 99–126)	100 (13.4; 74–130)

#### Stimuli

For the social condition, the visual stimuli comprised 24 standardized images of facial expression taken from the Mac Brain/NimStim Face Stimulus Set^1^ (Tottenham et al., 2009). There were eight faces (four male and four female) for each of three emotions (happy, fearful, and neutral). The faces with the highest validation ratings were selected. For the non-social condition, 24 different images from the International Affective Picture System (IAPS; Lang et al., 1995) depicted affective scenes and objects; there were eight pictures for each of three emotions (happy, fearful, and neutral). None of the non-social images contained human faces. The happy IAPS stimuli included the following image numbers: 1920, 5200, 5480, 5760, 5910, 7260, 7330, and 8502. The fearful IAPS stimuli included the following image numbers: 1120, 1200, 1525, 1930, 5971, 6230, 9480, and 9600. The neutral IAPS stimuli included the following image numbers: 7006, 7010, 7035, 7080, 7090, 7150, 7170, and 7235. One hundred college students (half female) have rated each of the images in the IAPS set for valence, arousal, and dominance; thus, norms are available for each image in the IAPS manual (Lang et al., 1995). Consistent with Baumgartner et al.’s (2006) study, and our previous study (Järvinen-Pasley et al., 2010a), a pilot study to facilitate selecting the visual non-social stimuli was carried out. Forty typical adults, who did not participate in the present experiments, identified the valence, and used a nine-point Likert-style scale to rate the intensity of a large set of IAPS stimuli. The piloting phase included 45 non-social images (15 images per emotion). The images that most reliably conveyed the intended emotion and had the greatest intensity became the test stimuli (except in the case of neutral affect, for which the images associated with the lowest intensity were selected). Overall, the valence and arousal ratings from our pilot study were similar to those that Lang et al. (1995) found in adults. Given that the IAPS stimuli have a relatively limited number of non-aversive non-social images that do not contain human faces, it was necessary to include images containing animals within the non-social category. The rationale for including NimStim face stimuli and IAPS images as stimuli was that, it is well established that these two classes of affective stimuli elicit differential neural responses (e.g., Meyer-Lindenberg et al., 2005), and thus differences in autonomic responsivity were also expected. Both the face and non-social stimuli were standardized for brightness and contrast using Matlab (MathWorks, Inc.).

#### Procedure

The experiment was conducted in a quiet room. Participants sat in a comfortable chair in a well-lit room, 130 cm away from a TFT monitor (screen resolution of 1680 × 1050 pixels). The experiment had two parts: a passive version, which was always administered first (for psychophysiological measurement), and an active task, during which participants made affect identification judgments. The stimuli were presented on a desktop computer running Matlab (Mathworks, Inc.), which delivered a digital pulse embedded in the recording at the onset of each stimulus. To measure physiological responses, after a fixation cross for 1000 ms, each stimulus was presented for 5000 ms, separated by an interstimulus interval (ISI) of 9000 ms (blank screen) to allow enough time for autonomic activity to return to near baseline levels. The stimuli were randomized with respect to both stimulus type (social/non-social) and affect valence (fearful/happy/neutral), and preceded by a blinking fixation cross. Participants were told that pictures of faces and scenes/objects/animals would appear on the screen in a random order. For the passive task, participants were only instructed to look at the pictures carefully, while remaining as quiet and still as possible. For the active task, participants were shown the same stimuli again with a briefer exposure than in the passive task (2000 ms), and asked to identify the emotion shown by the stimuli. Prior to the onset of the active task, the experimenter then showed the response screen to the participant, which listed the three possible emotions to ensure that the participant understood each of the emotion options (scary/scared, happy, and “no emotion” as a label for neutral). The participants responded verbally, and the experimenter operated the computer keyboard on the participant’s behalf. After each behavioral trial, participants were asked to identify the gender of the faces, and the scene/object name to ensure attention was maintained throughout the task. No group differences were observed in post-trial questions (*p* = 0.96).

#### ANS measures and statistical analyses

For both experiments 1 and 2, EDA and electrocardiogram (ECG) measures were recorded during the passive stages of the experiments using BioPac MP150 Psychophysiological Monitoring System (BioPac systems, Inc., Santa Barbara, CA, USA) at a sampling rate of 1000 Hz. Ag/AgCl electrodes where applied to the skin with an isotonic NaCl electrolyte gel placed on the index and middle distal phalanges of the participant’s non-dominant hand to record EDA. ECG was recorded with two electrodes, one attached to the right forearm, and the other attached to the left ankle, below the true ankle joint, located on the calcaneus. Recording sessions for each modality were preceded by a 5-min period of rest for the participant, during which baseline measurements were established. During the experiment, stimulus onsets were marked with trigger codes, embedded into the recordings.

All ANS measures were analyzed 7 s subsequent to stimulus presentation on a trial-by-trial basis, in comparison to a 3 s pre-stimulus baseline. All measured signals (EDA and ECG) were qualitatively inspected for the presence of artifacts. All trials containing outliers, which exceeded 2.5 SDs above or below the mean, were removed from analyses. Mean HR and inter-beat interval (IBI) measures were calculated from the raw ECG signal. After defining the R peak of the heart beat cycle, we calculated the mean and standard deviation of the inter-beat interval (sdIBI) to assess variation in heartbeats for each condition of the experiment. Quantification of the mean IBI is used in conjunction with mean HR since it is a more sensitive and direct measure of parasympathetic and sympathetic system activation (Bernston et al., 1995). Measurement of the sdIBI mirrors the more commonly seen calculation of root mean square of successive differences in heart beat intervals (RMSSD), and thus high frequency heart rate variability (HRV) information corresponding to vagal influence and parasympathetic activity (Bernston et al., 2005; Mendes, 2009). The standard deviation of IBI was chosen as an indirect time-domain measure of HRV due to the randomized and, relatively, quick presentation of stimuli.

All psychophysiology data were analyzed using R (R Development Core Team, 2008), and the R package *nlme* (Pinheiro et al., 2012). A linear mixed-effects model approach was used to assess mean tonic EDA, mean HR, sdIBI, and mean IBI measures taking into account random effects due to individual differences between-participants, autocorrelations between subsequent trials measurements, and possible covariates (age and accuracy on the behavioral task). Fixed effects in our models included group (WS/TD), condition (social/non-social), emotion (fearful/happy/neutral or sad), and the trial number (48 levels). For clarity, we report the *F* and *p* values of the Type III Sum of Squares tests of fixed effects from our models. We do not report degrees of freedom in our comparisons since these calculations in linear mixed-effects models are simply approximations. All pair-wise comparisons were Bonferroni corrected. The normality and homogeneity assumption for the linear mixed-effects models was assessed by examination of the distribution of residuals.

### Results

#### Behavioral affect identification

**Figure 1:**
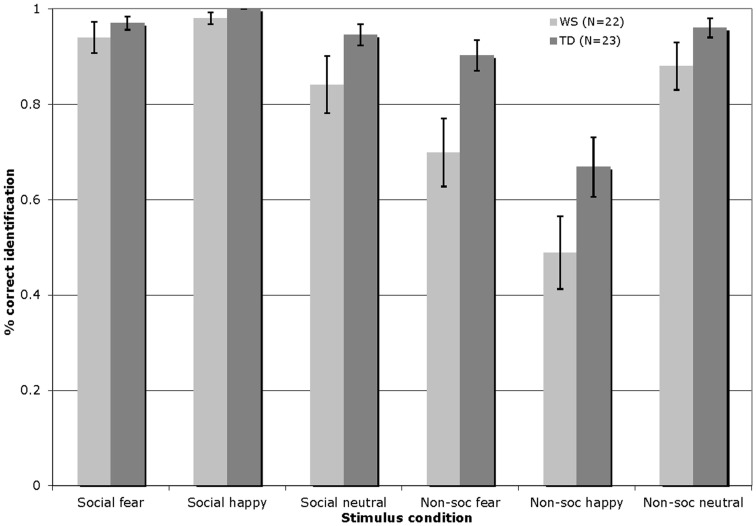
**Accuracy of visual affect identification for individuals with WS and TD, across the happy, fearful, and neutral categories for both social and non-social stimuli in Experiment 1**. (Total number of trials per affect category = 8; Error bars represent ± 1 standard error mean, SEM).

Figure 1 displays the percentage of correct identifications within each affect category (fearful/happy/neutral) across the social and non-social stimulus conditions in Experiment 1 for participants with WS and TD (total number of trials per affect category = 8).

The visual affect identification data were analyzed by a 2 × 3 × 2 repeated-measures ANOVA, with the condition (social/non-social) and emotion (fearful/happy/neutral) entered as within-participants factors, and group (WS/TD) as a between-participants factor. This analysis revealed a significant main effect of condition [*F*(1, 43) = 53.52, *p* < 0.001], reflecting higher levels of performance overall with the social stimuli; a significant main effect of emotion [*F*(2, 86) = 7.49, *p* = 0.001], with higher levels of identification of the fearful and neutral as compared to the happy stimuli; and a significant effect of group [*F*(1, 43) = 12.73, *p* = 0.001], indicating that the TD group outperformed those with WS. In addition, a condition by group interaction emerged [*F*(1, 43) = 4.54, *p* < 0.04]. Follow-up Bonferroni corrected *t*-test analyses (significance set at *p* ≤ 0.025) showed that the interaction effect arose due to the fact that while both groups exhibited similar levels of performance with the social stimuli [*t*(43) = −1.84, *p* = 0.07], the performance of the WS group with the non-social stimuli was significantly lower than that of the TD group [*t*(43) = −3.38, *p* < 0.01]. More specifically, the TD group outperformed the WS group in the identification of fearful non-social stimuli [*t*(43) = −2.63, *p* = 0.01]. There were no other between group differences (all *p* > 0.07). Pair-wise *t*-tests comparing within-group performance across the social and non-social conditions showed that both groups of participants exhibited higher overall identification performance with the social as compared to the non-social stimuli [WS: *t*(21) = 5.33, *p* < 0.001; TD: *t*(22) = 5.30, *p* < 0.001].

An error analysis was then carried out. The dependent measure, i.e., the proportion of errors committed, was computed separately for the individual social and non-social affect categories. The frequency of incorrect social/non-social emotion identification responses was divided by the total number of errors committed for the targeted affect category inclusive of both social and non-social stimuli. For example, a participant who erroneously labeled four neutral faces as “happy” and two neutral faces as “scary” out of a total of 10 errors within the neutral affect category inclusive of social and non-social trials, yielded an error proportion of 0.40 for incorrect happy labels, and 0.20 incorrect scary labels, within the neutral social targets. Please note that the total number of trials inclusive of social and non-social conditions is 48. In order to explore any systematic patterns in participants’ incorrect responses to the visual neutral stimuli, a condition (social/non-social) × error type (fearful/happy) × group (WS/TD) mixed ANOVA was conducted. Condition was included as an independent variable as well as error type, i.e., the incorrect emotion provided in place of the targeted affect. No significant results emerged for the neutral targets (all *p* > 0.05). For happy targets, significant main effects of condition [*F*(1, 43) = 541.92, *p* < 0.001] and error type [*F*(1, 43) = 591.28, *p* < 0.001] emerged. More errors were made in identifying happy non-social (*M* = 0.46) as compared to happy social (*M* = 0.004) stimuli. Additionally, happy stimuli were more frequently incorrectly identified as neutral (*M* = 0.46) than fearful (*M* = 0.001). Finally, a condition × error type interaction reached significance [*F*(1, 43) = 553.37, *p* < 0.001], with happy non-social (*M* = 0.92) as compared to happy social (*M* = 0.006) stimuli being more frequently incorrectly identified as neutral [*t*(44) = 23.72, *p* < 0.001]. Bonferroni correction was employed for rest of the *t*-test analyses (significance set at *p* ≤ 0.025). Participants mislabeled happy non-social images as neutral (*M* = 0.92) at a greater rate than misidentifying happy social stimuli as fearful [*M* = 0.003; *t*(44) = 24.48, *p* < 0.001]. For fearful affective targets, significant main effects of condition [*F*(1, 43) = 8.94, *p* < 0.01], error type [*F*(1, 43) = 27.34, *p* < 0.001], and group [*F*(1, 43) = 8.62, *p* < 0.01] were found. Incorrect identification of fearful stimuli occurred at a greater rate for non-social (*M* = 0.24) as compared to social (*M* = 0.09) images, and more errors were made in identifying fearful images as neutral (*M* = 0.28) than happy (*M* = 0.05). Participants with WS made more errors in identifying fearful stimuli as compared to the TD group (*M* = 0.21, *M* = 0.12, respectively). A significant interaction of condition × error type was observed [*F*(1, 43) = 27.34, *p* < 0.001], with fearful non-social stimuli being more frequently incorrectly identified as neutral (*M* = 0.44) than happy [*M* = 0.04; *t*(44) = 5.12, *p* < 0.001]. Finally, fearful non-social (*M* = 0.46) as compared to social (*M* = 0.12) stimuli were more frequently mislabeled as neutral [*t*(44) = 3.46, *p* = 0.001].

#### Psychophysiological results

**Figure 2:**
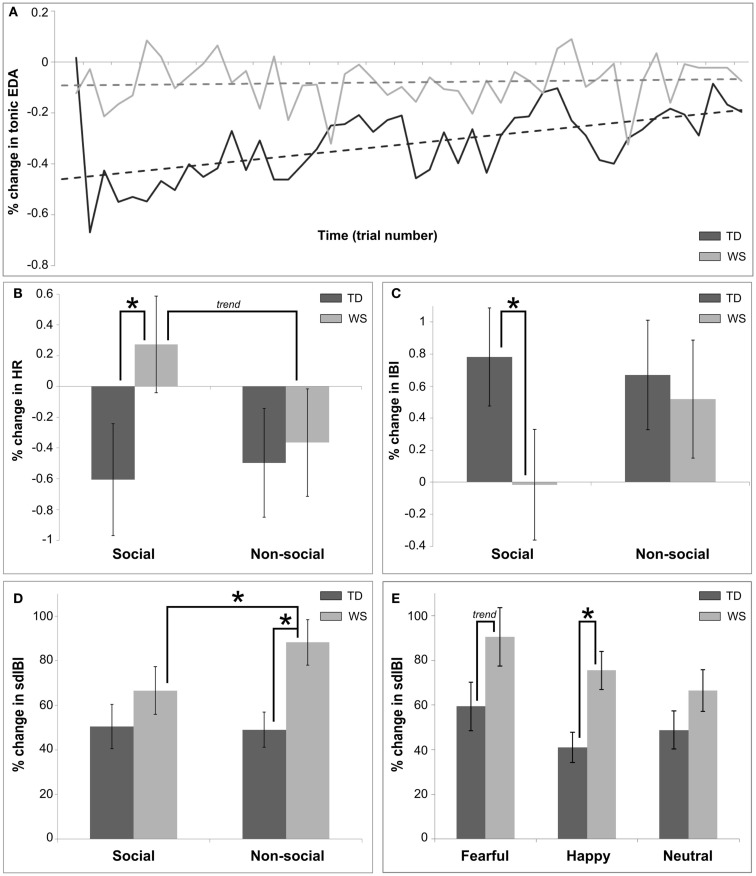
**Summary of main findings utilizing autonomic measures for Experiment 1, the visual modality**. **(A)** Shows the differences in overall habituation over time between TD and WS participants, calculated as a percentage change between baseline (3 s pre-stimulus) and post stimulus (7 s after) in the mean tonic EDA. **(B)** Displays differences between social and non-social conditions in TD and WS participants, calculated as a percentage change between baseline and post stimulus in the mean HR. **(C)** Presents the differences between social and non-social conditions in TD and WS participants, calculated as a percentage change between baseline and post stimulus in the mean IBI. **(D)** Displays differences between social and non-social conditions in TD and WS participants, calculated as a percentage change between baseline and post stimulus in the sdIBI. **(E)** Displays differences between affective stimuli in TD and WS participants, calculated as a percentage change between baseline and post stimulus in the sdIBI.

Figure 2 describes the overall pattern of tonic EDA in WS and TD groups over trial number within the visual modality. The TD group showed a greater percentage change in tonic EDA as compared to the individuals with WS (*F* = 14.11, *p* < 0.001). A significant effect of trial number was found (*F* = 14.69, *p* = 0.0001), suggesting that habituation to the stimuli occurred over time during the experiment. However, there was a group by trial interaction, such that the habituation effect was predominantly observed in TD participants (*F* = 8.81, *p* = 0.003; see Figure 2A). Finally, there was no effect of emotion on tonic EDA (*p* > 0.05).

Analysis of the mean HR also revealed significant group differences, with a greater decrease in HR in the TD group as compared to those with WS (*F* = 4.32, *p* = 0.04). A main effect of trial number indicated an overall reduction in percentage change of HR decreased over time in both groups (*F* = 9.51, *p* = 0.002). Emotion modulated changes in HR (*F* = 4.6, *p* = 0.01), and pair-wise comparisons revealed that fearful stimuli elicited on average a greater decrease in HR in comparison to both happy and neutral emotional stimuli for both groups (both *p*’s < 0.05). While there was no main effect of condition, a group by condition interaction emerged (*F* = 4.48, *p* = 0.034; see Figure 2B). Further exploration showed that this interaction was in part due to the differences between the WS and TD participants in the social condition only (*p* < 0.05), with a decrease in HR for the TD group, yet an overall increase in HR for individuals with WS when viewing faces.

Inter-beat interval measurement is based upon an analysis of individual heartbeat cycles, therefore changes in mean IBI between the baseline-level and that after stimulus onset is a sensitive method of quantifying heart beat acceleration/deceleration. The analysis of the mean IBI revealed significant main effects of group (WS change in mean IBI greater than that of TD group; *F* = 5.7, *p* = 0.021), emotion (*F* = 7.07, *p* < 0.001; larger change in mean IBI for fearful compared to other emotions, both *p*’s < 0.05), and trial number (reduction of change in mean IBI over time; *F* = 12.58, *p* < 0.001). There was an interaction effect between emotion and condition (*F* = 5.48, *p* < 0.001), driven by a greater change in mean IBI for the fearful non-social condition in comparison to other non-social stimuli (both *p*’s < 0.05). Further, there was a group by condition interaction (*F* = 5.98, *p* = 0.015), with individuals with WS displaying approximately zero mean change in IBI for the social condition, contrasting with a greater change in controls (*p* < 0.05; Figure 2C).

Figure 2 displays the sdIBI across conditions and groups (Figure 2D) and emotion across group (Figure 2E). The IBIs of participants with WS were more variable than those of the TD group (*F* = 20.17, *p* < 0.0001). A main effect of condition indicated that non-social conditions produced overall greater variability (*F* = 4.69, *p* = 0.03), while an interaction between group and condition highlighted that this increase in variability was attributable to the WS group (*F* = 5.83, *p* < 0.02). Further analyses of this interaction revealed increased sdIBI in the WS group as compared to participants with TD in the non-social condition, and increased sdIBI for the non-social relative to the social condition in WS only (all *p*’s < 0.05; Figure 2D). A main effect of emotion was also found (*F* = 6.76, *p* = 0.001), suggesting that greater variability in IBI was observed for stimuli eliciting fearful as compared to happy and neutral emotions (all *p*’s < 0.05). Finally, there was a trend for an interaction between group and emotion such that the IBIs of individuals with WS were greater in variance than those of the TD group’s (*F* = 2.88, *p* < 0.06; Figure 2E), due to an ampler variation in response to happy (*p* < 0.05) and fearful emotional stimuli (*p* = 0.07).

### Brief discussion

The goal of experiment 1 was to compare the processing of social and non-social visually presented affective stimuli at the levels of behavior and ANS responsivity in individuals with WS contrasted with a TD group. The main behavioral result showed that while individuals with WS performed at a comparable level to the TD comparison group in processing emotional faces, they were significantly poorer at identifying affect in non-social images. This result is in line with earlier studies (e.g., Järvinen-Pasley et al., 2010a,b) suggesting that WS is characterized by a bias toward social information manifesting as superior processing of social over non-social stimuli. An analysis of participants’ error patterns further revealed that, in line with the existing literature, participants with WS showed difficulties in identifying fearful visual emotion as compared to the comparison group (e.g., Meyer-Lindenberg et al., 2005; Plesa Skwerer et al., 2009; Järvinen-Pasley et al., 2010c). Finally, in WS only, VIQ was positively correlated with affect identification in the social condition, suggesting parallel development of emotion recognition and linguistic capacities in our WS population. Indeed, it remains to be studied whether stronger linguistic abilities may enhance emotional understanding. This may be of specific importance to clinical populations with known emotional problems, e.g., autism.

An analysis of the ANS indices within the visual domain revealed many between group differences resulting from cardiovascular measures. First, it is noteworthy that individuals presenting with supravalvular aortic stenoses and hypertension, as is often the case in WS, do not show evident changes in raw ECG for quantification of the R peak and IBI, even if abnormalities in the QRS complex can be observed (see Dimopoulos et al., 2012, for an overview). Moreover, the use of change scores weighted at the baseline period rules out spurious differences in cardiovascular functioning. Notwithstanding these differences, the WS group showed an overall increase in mean HR for social stimuli in comparison to TD participants. This stands in contrast to previous publications demonstrating HR deceleration to dynamic face images in WS relative to controls (Plesa Skwerer et al., 2009). Furthermore, WS individuals showed decreased variation in heart periods (sdIBI) to faces in comparison to objects/scenes, similar to our behavioral results suggesting a greater emotional reactivity to faces in comparison to objects/scenes, since cardiac vagal reactivity is reduced for emotionally arousing stimuli. In the non-social condition, higher sdIBI can be thought of as indicating feelings of calm, equanimity, and control (Porges, 2007), yet in our WS population this possibly could be interpreted as attenuated emotional arousal in the non-social condition. Indeed, the significantly increased sdIBI and reduced performance in the non-social condition for WS individuals in contrast to controls, suggests diminished arousal in WS to non-face objects and scenes across all emotions. Similarly, an ampler response to happy and fearful stimuli for WS in comparison to TD individuals is not consistent with an interpretation of increased calmness, control, and so forth to happy or fearful face/object stimuli, but rather with the diminished arousal to non-face objects. Deterioration in performance observed in individuals with WS in relation to TD participants supports this view.

An analysis of the tonic EDA signal between the WS and TD groups revealed one striking difference. Whereas participants with TD showed a classical habituation effect over time, that is, reactivity to stimuli was attenuated as the trials progressed (see Figure 2A), individuals with WS showed patterns of responsivity that remained stable over time. Thus, our participants with WS did not habituate, regardless of their overall changes in EDA, in relation to TD individuals. This suggests significantly different rates of familiarization to affective visual stimuli across groups.

## Experiment 2: Vocalizations vs. Melodies: ANS Sensitivity to Auditory Social and Non-Social Affective Stimuli

### Material and methods

#### Participants

Twenty individuals with WS (seven males) were recruited and screened as described under Experiment 1. All of these participants also participated in Experiment 1. Twenty-six TD comparison individuals (10 males) were also recruited and screened as described above. Twenty-two of these individuals also participated in Experiment 1, with psychophysiological analyses conducted on the same participants used for analysis in Experiment 1. Participants were administered a threshold audiometry test using a Welch Allyn AM232 manual audiometer, which was calibrated to ANSI s.3.21 (2004) standards. Auditory thresholds were assessed at 250, 500, 750, 1000, 1500, 2000, 3000, 4000, 6000, and 8000 Hz, monaurally. The hearing of all participants included in the study was within the normal range.

Table 2 shows the demographic characteristics of the final sample of participants with WS and TD. The participants differed in terms of CA [*t*(44) = 3.14, *p* = 0.003] with the WS group being older than the TD group. The TD group scored significantly higher on VIQ, PIQ, and FSIQ (all *p* < 0.001). Pearson correlations exploring the potential contributions of CA, VIQ, PIQ, and FSIQ to task performance, showed no significant associations (WS: all *p* > 0.43, TD all *p* > 0.09).

**Table 2:** **Mean characteristics of the participant groups (SD; range in parentheses) in Experiment 2**.

	CA (SD; range)	VIQ (SD; range)	PIQ (SD; range)	FSIQ (SD; range)
WS (*n* = 20)	27.2 (8.5; 13–46)	67 (8.4; 55–83)	63 (6.2; 53–72)	66 (7.2; 56–84)
TD (*n* = 26)	21.2 (4.3; 18–41)	113 (9.4; 89–128)	115 (7.4; 99–127)	115 (6.9; 99–126)

#### Stimuli

For the social condition, the visual stimuli comprised 24 segments of non-linguistic vocal sounds (2–3 s/segment) taken from the “Montreal Affective Voices,” a standardized set of vocal expressions without confounding linguistic information (freely)^2^. There were eight segments for each of three emotions (happy, fearful, and sad). The non-social condition included 24 segments of novel, normed musical pieces, eight segments eliciting each of three possible emotions (fearful, happy, sad). The segments of unfamiliar emotionally evocative music have been specifically composed by Marsha Bauman of Stanford University for studies to examine musical abilities in WS (see also Järvinen-Pasley et al., 2010a). These segments have been pre-tested in typical adults to confirm that they convey happy, fearful, or sad emotion with >95% accuracy.

#### Procedure

As in Experiment 1, the study had two phases: a passive version, which was always administered first (for psychophysiological measurement), and an active task, during which participants made affect identification judgments. EDA and ECG were recorded as described under Experiment 1, and the experimental apparatus were the same. A fixation cross was presented for 1000 ms before presentation of the stimulus, which were randomized with respect to both stimulus type (social/non-social) and affect valence (fearful/happy/sad). The duration of the auditory clips, presented at the onset of a 5000 ms blank screen, were slightly variable as described above, with human voices presented for an average of 1353 ± 642 ms, and music clips presented for 1354.67 ± 538.93 ms on average, since we required the stimuli to be as natural as possible. For example, an extended period of laughter might not elicit a happy emotional response. The inter-trial interval was 9000 ms, to again allow time for the autonomic levels to return to near baseline. Participants were told that they would hear short sounds that would either be a voice or music. For the passive task, participants were only instructed to listen to the sounds carefully while attending to a monitor displaying a fixation cross, and staying as quiet and still as possible. For the active task, participants were played the stimuli again sequentially, and asked to identify the emotion elicited by each sound. A response screen contained the words “scared/scary,” “happy,” and “sad.” The participants responded verbally, and the experimenter operated the computer keyboard on the participant’s behalf.

### Results

#### Behavioral affect identification

**Figure 3:**
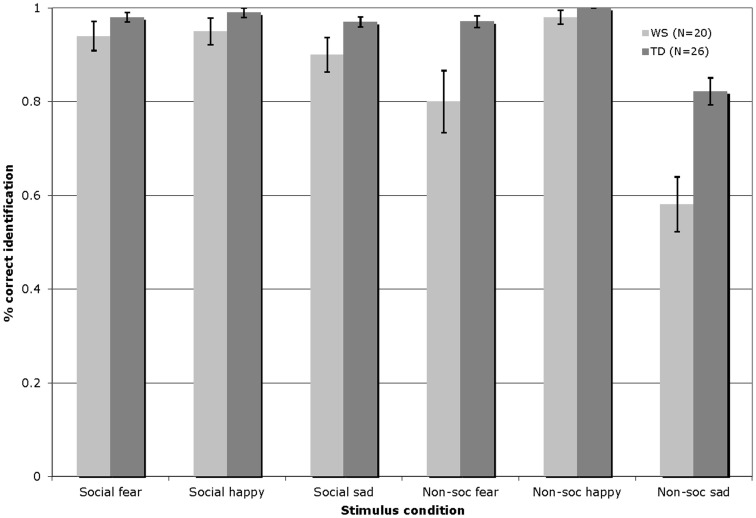
**Accuracy of auditory affect identification for individuals with WS and TD, across the happy, fearful, and sad categories for both social and non-social stimuli in Experiment 2 (Total number of trials per affect category = 8; Error bars represent ± 1 standard error mean, SEM)**.

Figure 3 displays the percentage of correct identifications within each affect category (fearful/happy/sad) across the social and non-social stimulus conditions in Experiment 2 for participants with WS and TD (total number of trials per affect category = 8).

The auditory affect identification data were analyzed by a 2 × 3 × 2 repeated-measures ANOVA, with the condition (social/non-social) and emotion (fearful/happy/sad) entered as within-participants factors, and group (WS/TD) as a between-participants factor. This analysis revealed a significant main effect of condition [*F*(1, 44) = 42.36, *p* < 0.001], reflecting higher levels of performance overall with the social stimuli; a significant main effect of emotion [*F*(2, 88) = 48.34, *p* < 0.001], reflecting overall highest levels of identification of the happy as compared to the fearful and sad stimuli, and higher levels of identification of fearful, as compared to the sad stimuli; and a significant effect of group [*F*(1, 44) = 13.84, *p* = 0.001], indicating that the TD group outperformed those with WS. In addition, a condition by group interaction [*F*(1, 44) = 9.85, *p* = 0.003], a group by emotion interaction [*F*(1, 88) = 6.33, *p* = 0.003], and a condition by emotion by group interaction emerged [*F*(1, 88) = 4.26, *p* < 0.02]. Follow-up Bonferroni corrected *t*-test analyses (significance set at *p* ≤ 0.0125) showed that the interaction effects emerged due to the fact that while both groups exhibited similar levels of performance with the social happy [*t*(44) = −1.45, *p* = 0.15] and social scary [*t*(44) = −1.51, *p* = 0.14] the TD group outperformed their counterparts with WS in identifying social sad [*t*(44) = −2.06, *p* = 0.05] stimuli. Within the non-social domain, again both groups yielded similar accuracy in recognizing non-social happy stimuli [*t*(44) = −1.96, *p* = 0.06)] while the TD group outperformed their counterparts with WS in identifying both non-social fearful [*t*(44) = −2.87, *p* = 0.006], and non-social sad [*t*(44) = −3.98, *p* < 0.001] stimuli. Relative to those with WS, the TD group showed higher affect identification performance overall with both the social [*t*(44) = −2.15, *p* = 0.04] and the non-social [*t*(44) = −4.03, *p* < 0.001] stimuli. Pair-wise *t*-tests comparing within-group performance across the social and non-social conditions showed that whereas the WS participants exhibited similar identification performance across the happy social and non-social stimuli [*t*(19) = −0.85, *p* = 0.41] they showed higher identification performance with the fearful [*t*(19) = 2.73, *p* = 0.013)] and sad [*t*(19) = 5.46, *p* < 0.001)] social, as compared to the non-social fearful and sad stimuli. Overall, performance was significantly higher in the social, as compared to the non-social condition [*t*(19) = 5.29, *p* < 0.001]. Within-condition comparisons revealed that while the WS group showed similar performance across all emotions within the social condition {happy vs. fearful [*t*(19) = 0.32, *p* = 0.76], happy vs. sad [*t*(19) = 1.80, *p* = 0.09], fearful vs. sad [*t*(19) = −0.96, *p* = 0.35]}, with the non-social music stimuli, performance was higher with the happy, as compared to both the fearful [*t*(19) = 2.75, *p* = 0.013] and sad [*t*(19) = 6.93, *p* < 0.001] stimuli, and performance was higher with the fearful as compared to the sad non-social stimuli [*t*(19) = 3.77, *p* = 0.001]. By contrast, the TD participants performed similarly across the social and non-social conditions with the happy [*t*(25) = −1.0, *p* = 0.33] and fearful [*t*(25) = 0.68, *p* = 0.50] stimuli, while performance was significantly higher with the sad social as compared to sad non-social stimuli [*t*(25) = 4.69, *p* < 0.001]. Again, the performance of the TD group was significantly higher overall in the social, as compared with the non-social condition [*t*(25) = 3.40, *p* = 0.002]. Within-condition comparisons revealed that similarly to the WS group, the TD group showed comparable performance across all emotions within the social condition {happy vs. fearful [*t*(25) = 0.52, *p* = 0.61], happy vs. sad [*t*(25) = 1.69, *p* = 1.0)] fearful vs. sad [*t*(25) = −0.81, *p* = 0.43]}. With the non-social music stimuli, whereas performance was similar across the happy vs. the fearful music stimuli [*t*(25) = 2.29, *p* = 0.03], identification was higher with the happy vs. sad [*t*(25) = 6.19, *p* < 0.001], and scary vs. sad [*t*(25) = 5.37, *p* < 0.001] stimuli.

An error analysis was then carried out as described under Experiment 1. In order to explore any systematic patterns in participants’ incorrect responses to the sad auditory stimuli, a condition (social/non-social) × error type (fearful/happy) × group (WS/TD) mixed ANOVA was conducted, analogous to the analysis within the visual domain. Main effects of condition [*F*(1, 44) = 69.27, *p* < 0.001] and error type [*F*(1, 44) = 44.11, *p* < 0.001] emerged, with more errors being made in identifying sad affect when the stimuli were non-social (*M* = −0.38) as compared to social (*M* = 0.07) in nature. In addition, a significant interaction between condition and error type was observed [*F*(1, 44) = 28.39, *p* < 0.001]. Bonferroni corrected *t*-tests (with significance set at *p* ≤ 0.025) showed that participants made more errors in identifying sad stimuli as happy when it was non-social (*M* = 0.64) as compared to social [*M* = 0.09; *t*(45) = 7.42, *p* < 0.001] in nature. Finally, participants incorrectly identified sad non-social stimuli as happy (*M* = 0.64) more frequently than misidentifying sad non-social stimuli as fearful [*M* = 0.10; *t*(45) = 6.72, *p* < 0.001]. For happy targets, a significant main effect of group emerged [*F*(1, 44) = 4.72, *p* < 0.05], with participants with WS showing a higher overall error rate to the TD group (*M* = 0.06, *M* = 0.01, respectively). Finally, for fearful targets, the identification of non-social (*M* = 0.04) as compared to social (*M* = 0.16) stimuli resulted in greater rate of errors [*F*(1, 44) = 8.47, *p* < 0.01].

#### Psychophysiological results

**Figure 4:**
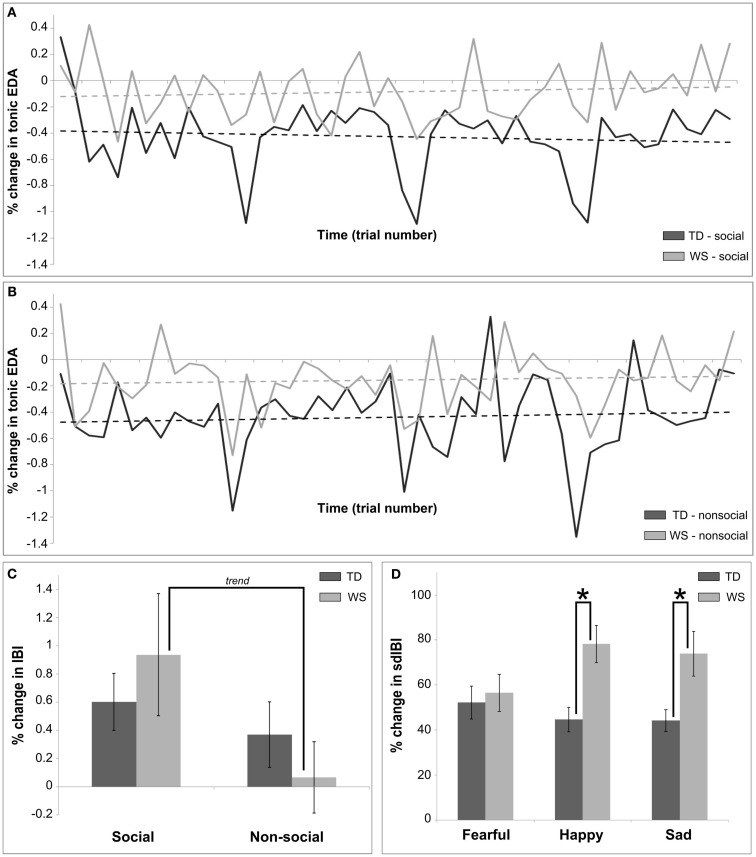
**Summary of main findings utilizing autonomic measures for Experiment 2, the auditory modality**. **(A)** Shows the differences in habituation over time in the social condition between TD and WS participants, calculated as a percentage change between baseline and post stimulus in the mean tonic EDA. **(B)** Shows the differences in habituation over time in the non-social condition between TD and WS participants, calculated as a percentage change between baseline and post stimulus in the mean tonic EDA. **(C)** Presents the differences between social and non-social conditions in TD and WS participants, calculated as a percentage change between baseline and post stimulus in the mean IBI. **(D)** Displays differences between affective stimuli in TD and WS participants, calculated as a percentage change between baseline and post stimulus in the sdIBI.

As within the visual modality, the TD participants displayed a larger percentage change in tonic EDA as compared to those with WS (*F* = 10.83, *p* = 0.002). There were no other significant main effects (all *p*’s > 0.05). There was a condition by trial number interaction (*F* = 6.12, *p* < 0.02), however, this is better explained by a three-way interaction with group (*F* = 4.57, *p* = 0.03; Figures 4A,B), highlighting that changes in tonic EDA were attenuated in participants with WS in comparison to the TD group for both social and non-social stimuli, but that the pattern of social and non-social stimuli in controls was highly variable over time. Moreover, a three-way interaction between emotion, condition and trial number (*F* = 3.4, *p* = 0.03) suggested that, for both groups, as trials progressed non-social stimuli elicited larger changes in tonic EDA than social stimuli, with this interaction predominantly driven by variable patterns of response to emotional stimuli over time.

An analysis of mean HR activity revealed a main effect of emotion (*F* = 4.19, *p* < 0.02), due in part to a trend for greater HR deceleration for stimuli eliciting sad emotions compared to happy emotions (*p* < 0.09). Moreover, the manipulation of social/non-social conditions produced a significantly greater decrease in HR for social voice stimuli in comparison to non-social music stimuli (*F* = 4.15, *p* = 0.04). A main effect of trial number (*F* = 16.24, *p* = 0.0001) suggested that the overall magnitude of the change in mean HR decreased over the duration of the experiment. No other comparison of mean HR reached significance (all *p*’s > 0.05).

An analysis of the percentage change in mean IBI revealed main effects of condition (greater change for social compared to non-social stimuli, *F* = 11.002, *p* < 0.001), emotion (*F* = 4.24, *p* = 0.015; pair-wise comparison: sad stimuli produced a greater change in IBI than happy, *p* = 0.09), and trial number (reactivity to all stimuli decreasing over time, *F* = 16.37, *p* = 0.0001). There was a modulation between groups by condition (*F* = 4.2, *p* < 0.04), such that social stimuli elicited a greater mean change in IBI when compared to non-social stimuli in the WS group only (pair-wise trend, *p* = 0.14; see Figure 4C). Finally, a condition by trial number interaction emerged (*F* = 5.31, *p* = 0.02), suggesting that reactivity to social stimuli was higher across groups during early stages of the experiment compared to non-social stimuli.

Across time, both groups showed a decrease in the variation in IBI (*F* = 4.38, *p* < 0.04). In addition, the participants with WS displayed patterns of greater sdIBI variation as compared to the TD group (*F* = 8.01, *p* < 0.01). Finally, Figure 4D highlights the changes in sdIBI across emotions between groups, with the two factors significantly interacting (*F* = 4.18, *p* = 0.015). Individuals with WS as compared to the TD group showed a greater change in IBI variability for happy and sad stimuli (both *p*’s < 0.05).

### Brief discussion

The main behavioral result from the auditory paradigm highlighted that the TD group outperformed those with WS in overall affect identification. However, within the social condition, individuals with WS showed comparable levels of accuracy to the TD group in identifying both happy and fearful vocalizations while showing specific deficits in the recognition of sad stimuli. This result is in partial agreement with our previous study, reporting unimpaired identification of positive, and impaired processing of negative, human vocalizations, relative to CA-matched TD individuals (Järvinen-Pasley et al., 2010b). Moreover, Haas et al. (2009) found significantly attenuated neural (event-related potential measures) and amygdala activity (shown by functional magnetic resonance imaging, fMRI) to sad facial stimuli in individuals with WS relative to TD, which may underlie the poorer behavioral processing observed in the current experiment. However, in the former study, all negative vocalizations including, e.g., screams, grunts, and gasps were grouped under a single valence label (negative). Thus, the results may have been specifically due to individuals with WS making proportionally more errors with sad stimuli as compared to the stimuli conveying the other valences. Within the non-social condition, the performance of WS group was significantly poorer to that of TD individuals, mainly due to poor recognition of negatively valenced (fearful and sad) music. Consistent with this, Dykens et al. (2005) documented that individuals with WS reported feeling happy or upbeat feelings in response to negatively valenced music pieces in about 33% of trials in their study. Interestingly, when this tendency was related to the individuals’ anxiety levels, it was found that positive feelings in response to negative music specifically characterized the persons with WS with the greatest fears and anxiety. Potential explanations offered by Dykens and coauthors included unawareness of negative affective states, or persistence toward positivity, in particularly anxious individuals with WS. However, in the present study, the performance of individuals with WS on the music trials was still above chance levels, indicating a globally preserved ability in affect recognition in our WS population to an essentially emotionally intangible stimulus class, i.e., music. An analysis of error patterns revealed only one between group difference, namely, individuals with WS made more errors as compared to the TD group in identifying both social and non-social happy stimuli.

Measures of autonomic activity revealed differences in WS and TD groups between reactivity to social (voice) and non-social (music) stimuli over time in the tonic EDA signal, such that individuals with WS exhibited reduced percentage change scores as compared to the TD participants. The three-way interaction observed is driven by the variable pattern of reactivity over time observed in the TD group (see Figures 4A,B). It is not plausible to claim, however, that it is similar to the lack of a habituation effect seen in the visual modality for WS, since across the conditions in the auditory modality, habituation of the tonic EDA was also not observed in the TD group.

Heart rate analysis revealed main effects of condition, emotion, and time. Across groups, decreases in HR were greater in relation to social as compared to non-social stimuli, and also for specific emotions (sad as compared to happy). Over time, HR deceleration was attenuated in both groups, caused by a converging baseline and post stimulus HR rate to a stable level throughout the experiment. This result suggests habituation in both groups to all stimuli. Further examination of sensitive IBI measures suggested differences in heart periods between social and non-social conditions in WS only, with a greater change in mean IBI seen for human vocalizations compared to music. This result stands in contrast to the visual modality, since, for visually presented images, heart period post stimulus deceleration was seen for non-social in comparison to social stimuli (Figure 2C), yet this finding failed to reach significance. Further, standard deviation of the IBI showed greater variability in response to happy and sad stimuli for individuals with WS in contrast to TD participants. The lack of increased variability to fear eliciting stimuli suggests increased vagal reactivity for happy and sad auditory clips in turn eliciting feelings of calm, equanimity, and control (Porges, 2007). This interpretation is not confounded by performance issues in WS since trial accuracy of participants was controlled for in our statistical model.

## General Discussion

The aim of the current multimodal study was to examine ANS sensitivity to both visually and aurally presented affect in individuals with WS, to elucidate one intriguing phenotypal WS characteristic: emotional reactivity and its relation to increased sociability. To our knowledge, no previous studies have addressed ANS indices in this syndrome in relation to auditory social (or non-social) information. In accordance with previous studies, individuals with WS exhibited higher levels of behavioral performance with the social as compared to non-social stimuli (e.g., Järvinen-Pasley et al., 2010a), within both visual and auditory modalities, while the TD comparison group showed similar levels of performance across the social and non-social conditions in both modalities, highlighting a “bias” toward social information in WS. The analysis of underlying ANS reactivity revealed striking differences between the groups. First, within the visual domain, ECG-derived analyses showed that while individuals with WS demonstrated HR acceleration for social stimuli, the TD group was characterized by deceleration, indexing significantly increased arousal to face stimuli in WS relative to TD. Second, individuals with WS demonstrated increased variation in heart periods (sdIBI) to non-face images in comparison to faces, suggesting a greater emotional reactivity to social in relation to non-social visual stimuli. Finally, the tonic EDA data indicated that, overall, whereas TD participants showed a classical habituation effect over time, that is, reactivity to stimuli was attenuated as the trials progressed, those with WS failed to habituate. In other words, individuals with WS showed significantly slower rate of familiarization to the affective visual stimuli as compared to their TD counterparts, to the point that there was no evidence of familiarization in the WS group. In the social domain, it is attractive to speculate that this aspect of ANS functioning may play a role in the increased attraction to faces and subsequent appetitive drive for affiliation in WS, as the lack of familiarization effect over time may also imply that such stimuli appear more novel for those with WS. However, further studies are needed to address this possibility. Furthermore, if WS individuals’ perception of their environment retains its originality, it is plausible that social encounters are determined by the frequency and novelty of such opportunities to interact.

The current results from the visual domain highlight new directions in which psychophysiological functioning may underlie the WS social phenotype. In contrast to the previous findings of Plesa Skwerer et al. (2009), which indicated HR deceleration to dynamic face images in individuals with WS relative to controls, we found relatively increased HR for faces in WS. One possible reason for this difference between the studies may be due to the nature of the stimulus features used, i.e., static in the present study as compared to dynamic in Plesa Skwerer et al.’s (2009) study. Moreover, the tonic EDA used in the current study provides a more global index of overall ANS functioning or tendency, rather than solely being a measure of ANS reactivity to particular stimuli, such as skin conductance spikes. Indeed, Riby et al. (2012) used tonic EDA as a measure of ANS sensitivity and found comparable reactivity to TD individuals when viewing live face stimuli in their sample of individuals with WS. On the other hand, phasic SCR spikes are most easily elicited by designs manipulating highly threatening or arousing situations (Lang et al., 1993; Lane et al., 1999), and as such this measure was not applicable to the current design. It is noteworthy that the individuals with WS tested by Plesa Skwerer et al. (2009) were younger in average (19 years) compared to the current sample (27 years). In the current study, age was further controlled for as a factor in the analyses, ruling out spurious differences in our sample affecting physiological measures. Similarly, time (trial number) was included as a factor in our models for both visual and auditory psychophysiological data. It is possible that this approach offers a new perspective on ANS functioning in WS, highlighting characteristics of the syndrome previously unseen, i.e., non-habituation to experimental stimuli. This result needs to be replicated with other measures, as well as other samples of participants with WS, to determine this effect as a feature of the WS social phenotype.

As mentioned above, the auditory paradigm produced differential results of ANS functioning relative to the visual experiment. This is not surprising in light of the fact that similar effects have been reported in the brain imaging literature, with visually presented affect resulting in more robust neural activation to aurally conveyed emotion (Adolphs, 2002, 2009). Specifically, while there is consensus of opinion that the amygdala represents a central subcortical structure involved in affective face processing, the literature is inconsistent regarding amygdala involvement in the processing of affective prosody (Scott et al., 1997; Anderson and Phelps, 1998; Adolphs and Tranel, 1999; Adolphs et al., 1999; Wildgruber et al., 2006). However, fMRI data illustrate the recruitment of the amygdala at least in the processing of fearful or threatening stimuli, regardless of their sensory modality (Dolan et al., 2001; Ethofer et al., 2006). The current results of ANS responsivity to auditory emotional stimuli in individuals with WS indicated decreased tonic EDA changes in response to both vocal and music stimuli in WS relative to the TD, suggestive of attenuated habituation to the stimuli; however, a similar pattern was also observed in the TD group.

Interestingly, the WS group was characterized by changes in heart periods for human vocalizations relative to music, suggesting reduced arousal to the social voice stimuli. The opposite pattern of reactivity was observed within the visual domain for individuals with WS, i.e., attenuated arousal to the non-social affective images relative to facial expressions of affect. These findings can be explained in terms of the polyvagal theory of cardiovascular functioning, which posits that the two branches of the vagus nerve support different behavioral systems. Specifically, the social engagement system is thought to be indexed by the activity of the myelinated vagus, through dampening of the HPA axis, in turn eliciting calm behavioral states (Porges, 2007). Our results suggest that, since heart beat deceleration has been linked with increased focused attention, essentially, human vocalizations were more engaging than our music stimuli. The absence of increased variability for fearful auditory stimuli in the WS group is in contrast to the aberrant reactions to fearful visual stimuli observed in such individuals in the current study, and with fMRI (Meyer-Lindenberg et al., 2005). Specifically, Meyer-Lindenberg et al. (2005) highlighted hypoactivation of the amygdala to fearful faces, and hyperactivation of the amygdala to fearful scenes, in individuals with WS relative to TD controls. However, valence-specific patterns of amygdala activation in response to face stimuli have also been reported in individuals with WS relative to TD controls (Haas et al., 2009). Consistent across modalities, individuals with WS as compared to the TD group showed significantly greater HR variability to happy stimuli, indexing increased vagal and thus parasympathetic involvement. Positive affective stimuli are more socially engaging, since they promote approach-related behaviors. Because the nuclei controlling muscles of the face and head are integrated with regulation of the myelinated vagus, there is a direct physiological link between HRV and social perception, interaction, and engagement. Furthermore, the control of the stapedius muscle in the middle ear, facilitating the recognition of human voices, is activated during raising of the eyelids, and thus also falls under control of the myelinated vagus (Porges, 2007). Hence, our experiment taps directly onto the preferred sensory channels for social engagement, interaction, and perception of information, suggesting atypical reactivity to positive affect in WS in both visual and auditory sensory systems.

The differences between ANS sensitivity in the visual and auditory modalities may reflect the relatively less clear affective cues provided by auditory, as compared to visual, stimuli, and associated differences in underlying neural processes (Adolphs, 2002, 2009). This pattern was also mirrored in behavioral performance, in that while accuracy remained stable across conditions and modalities for the TD participants, individuals with WS showed deterioration within the auditory domain, possibly due in part to the higher ambiguity of affect in auditory stimuli as compared to visual stimuli (cf. Adolphs, 2002, 2009). One possible explanation for the higher levels of performance of individuals with WS within the visual social domain, and the clearer pattern of ANS sensitivity to the visual social information, is that it may reflect the augmented salience of faces, and resultant attentional capture of such stimuli compared to non-social stimuli, in individuals with WS (Riby and Hancock, 2008, 2009). Using eye-tracking methodology, individuals with WS were found to fixate on people’s faces and specifically on the eye region for significantly longer than individuals with TD and autism (Riby and Hancock, 2008). In another study using face stimuli embedded in scenes, individuals with WS displayed spared attentional capture to faces when finding embedded faces, exaggerated fixation, and a reduced tendency to disengage from the face stimuli, relative to both TD and autism comparison groups (Riby and Hancock, 2009). Alternatively, the current result showing attenuated arousal to the social as compared to non-social stimuli within the auditory domain, and the opposite pattern within the visual domain, in WS, may also reflect the special status that musical stimuli may have for such individuals. Indeed, behaviorally, individuals with WS have been described as showing unusually intense emotional responses to music (Don et al., 1999; Levitin et al., 2004), while there are no such reports in relation to human vocalizations. It may thus be the case that including music as “non-social” stimulus does not provide an adequate or comparable contrast to human vocalizations, since music is potentially more interesting. Of relevance here are the fMRI findings of Levitin et al. (2003), who compared neural activation patterns to music and noise stimuli in individuals with WS. While TD individuals displayed greater activation in the superior temporal gyrus and the middle temporal gyri in response to music than to noise, the only region showing greater activation to music relative to noise stimuli in individuals with WS was the right amygdala. Thus, it may be that increased arousal to music stimuli in the current study relative to vocalizations reflects greater amygdala activation relative to other types of auditory stimuli in individuals with WS. Further investigations are warranted to clarify the neurobiological correlates of music vs. human affective voice processing in WS.

An alternative interpretation of HR reactivity that warrants discussion is linked to the characterization of the WS social phenotype, namely, that while individuals with WS display increased approach behaviors and overall increased social drive, they also exhibit a complex pattern of social anxieties (Dykens, 2003). In one model of social anxiety (Cook and Turpin, 1998), HR acceleration is considered as indicative of a defensive response to threatening stimuli, and has been directly linked to subjective feelings of fear and phobia (Elsesser et al., 2006), while HR deceleration is considered to reflect attentional orienting. Typically studies have reported increased HR acceleration in persons with high levels of social anxiety (e.g., Wieser et al., 2009). Thus, the profile of HR responsivity in individuals with WS in the current study appears to be consistent with those characterizing individuals with high social anxiety. However, the current stimuli cannot be considered as highly threatening due to their failure to elicit SCR spikes, which are characteristic of physiological reactivity to threatening/highly arousing social information. HR acceleration was observed, but this was not consistent for the fearful stimuli; rather, differences were selective across conditions and modalities. Consequently, it appears more plausible to interpret changes in HR as an index of arousal non-specific to fear.

In conclusion, the present study further extends the previous work on ANS functioning in WS, suggesting a more complex pattern indexed by EDA and cardiovascular reactivity. First, the lack of habituation observed in the EDA of individuals with WS suggests that visual information appears and remains affectively less familiar over time in WS, which might underlie two potentially intertwined aspects of the WS social phenotype – their increased desire to approach strangers and an unusually high attraction to faces (Mills et al., 2000; Riby and Hancock, 2008, 2009). Second, since our methods account for the known differences in normal heart functioning between WS and TD participants, we suggest that indices of cardiac activity can be just as informative when investigating physiological modulation to changes in affect. Further, differential patterns of responding at both behavioral and psychophysiological levels in WS (e.g., increased HR to faces) suggest acute autonomic reactivity to socially relevant stimuli (faces and voices) even if identification accuracy is lower relative to TD participants, which may underlie their increased emotional sensitivity and empathy as documented at the behavioral level (e.g., Tager-Flusberg and Sullivan, 2000). While it is tempting to highlight the increased appetitive drive for social interaction characterizing WS, our results suggest impairments in understanding emotion in non-social contexts. The overall pattern of increased ANS reactivity to social information in WS stands in sharp contrast to the profile reported for extraverted TD individuals as described in the introduction, and in fact resembles more of that associated with introversion. Future studies should further explore the unusual emotional sensitivity in WS in the context of specifically socially relevant information processing, to illuminate the basis of their unique social drive, together with the “peaks and valleys” characterizing the WS social phenotype across modalities.

## Conflict of Interest Statement

The authors declare that the research was conducted in the absence of any commercial or financial relationships that could be construed as a potential conflict of interest.

## Footnotes

^1^www.macbrain.org

^2^http://vnl.psy.gla.ac.uk/info.php?file=mav
